# Factors associated with health-related quality of life in patients with chronic liver disease in Korea: a cross-sectional study

**DOI:** 10.7586/jkbns.26.024

**Published:** 2026-08-27

**Authors:** Gum Mo Jung, Jihyon Pahn

**Affiliations:** 1 Division of Gastroenterology and Hepatology, Department of Internal medicine, Presbyterian Medical Center, Jeonju, Korea; 2 Department of Nursing, Jesus University, Jeonju, Korea

**Keywords:** Depression, Liver diseases, Quality of life, Health status, Uncertainty

## Abstract

**Purpose:**

This study aimed to identify factors associated with health-related quality of life (HRQoL) in patients with chronic liver disease and to provide foundational data for developing intervention programs.

**Methods:**

This cross-sectional study included 166 patients recruited from a general hospital in Jeonju, South Korea. Data were collected using structured questionnaires assessing depression, uncertainty in illness, and subjective health status and were analyzed using descriptive statistics, the independent t-test, one-way analysis of variance, Pearson correlation coefficients, and multiple regression analysis using SPSS version 23 program.

**Results:**

The mean HRQoL score was 3.19 ± 0.54 (range 1~5). Depression, uncertainty in illness, subjective health status, and education level were significant factors associated with HRQoL. Higher depressive symptom scores (β = -.24, *p* = .002) and greater uncertainty in illness (β = -.15, *p* = .044) were associated with lower HRQoL, whereas better subjective health status (β = .34, *p* < .001) and a higher education level were associated with higher HRQoL.

**Conclusion:**

These findings may inform intervention programs to improve HRQoL in patients with chronic liver disease by addressing depressive symptoms, uncertainty in illness, subjective health status, and educational needs.

## INTRODUCTION

Patients with chronic liver disease frequently experience persistent fatigue, pain, sleep disturbances, depression, anxiety, and limitations in physical functioning, all of which can impose a substantial burden on daily life and negatively affect health-related quality of life (HRQoL) [1]. Furthermore, because chronic liver disease requires long-term management and may progress to serious complications such as cirrhosis and hepatocellular carcinoma, patients often face considerable physical and psychological burdens [2].

In South Korea, liver disease ranks among the top 10 causes of death, with 7,787 deaths attributed to it in 2024 [3]. Particularly affecting economically active middle-aged adults in their 40s and 50s, liver disease holds significant importance as the 3rd most frequent cause of death, with the mortality rate in the 40s age-group reaching 12.2 deaths per 100,000 people in Korea [3]. Chronic liver disease is a heterogeneous group of conditions caused by various etiologies, including chronic hepatitis B and C, alcohol-related liver disease, and nonalcoholic liver disease, with differences in disease progression, clinical manifestations, and prognosis depending on the underlying cause. In addition, the severity and stage of the disease are important determinants of patients’ health outcomes and HRQoL [4].

HRQoL reflects patients' perceptions of the impact of illness and treatment on mental health, physical health, functional status, social relationships, and personal beliefs [5]. Therefore, understanding factors that influence HRQoL is essential for improving patient outcomes, particularly among individuals with chronic diseases. According to the Wilson and Cleary [6] model of HRQoL, biological and physiological factors influence symptom status, functional status, and general health perceptions, which ultimately affect overall HRQoL. In this framework, depression may impair psychological well-being and daily functioning, uncertainty in illness may hinder adaptation to disease-related events and increase psychological distress, and subjective health status reflects individuals’ overall evaluation of their health. Therefore, these factors may play important roles in shaping patients’ perceptions of health and ultimately influence HRQoL among patients with chronic liver disease.

Patients with chronic liver diseases, such as cirrhosis, typically report a lower quality of life than other patient groups [7]. Patients with chronic liver disease often experience fatigue, depression, and anxiety as part of their overall health status changes. Notably, depression rates are reported to be higher among patients with chronic liver disease than among other chronic disease groups [8], significantly impacting their daily and social life, and ultimately leading to diminished quality of life [9].

Patients with chronic liver disease often experience uncertainty in illness because the disease may progress without noticeable symptoms and its clinical course and prognosis are difficult to predict [2,10]. Furthermore, concerns regarding the potential development of cirrhosis, hepatocellular carcinoma, and other complications may increase uncertainty about future health outcomes. Previous studies have shown that higher levels of uncertainty are associated with lower quality of life [10].

Subjective health status serves as an integrated measure of an individual's overall health, effectively reflecting the current health status and predicting disease morbidity and mortality rates [11]. Quality of life in patients with chronic illnesses is influenced by subjective health status and disease progression, and subjective health status has been shown to significantly impact HRQoL [12].

Previous studies have investigated the factors influencing HRQoL in patients with chronic liver disease, including studies targeting patients hospitalized in gastroenterology departments [9], middle-aged men [13], and patients with hepatitis B [14], as well as studies using secondary data to examine HRQoL in Korean patients with chronic liver disease [12]. However, compared with other chronic conditions, research in this area remains relatively limited. Most previous studies have focused on examining individual factors affecting HRQoL separately, while relatively few studies have comprehensively considered multiple psychological and cognitive factors together. Because chronic liver disease is characterized by long-term disease management and substantial psychological burden, depression, uncertainty in illness, and subjective health status may interactively influence HRQoL. However, studies examining these variables within an integrated framework remain limited.

Therefore, this study aimed to identify the factors influencing the HRQoL of patients with chronic liver disease to enhance their HRQoL and provide foundational data for the development of effective intervention programs.

## METHODS

### 1. Study design

This study adopted a cross-sectional design. This descriptive survey aimed to identify factors influencing HRQoL in adults diagnosed with chronic liver disease. This study was reported in accordance with the STROBE (Strengthening the Reporting of Observational Studies in Epidemiology) statement, a guideline for reporting observational studies.

### 2. Participants

Eligible study participants were adults aged 19 years and older receiving treatment for chronic liver disease. In this study, chronic liver disease was defined as a physician-diagnosed liver disease lasting longer than 6 months. Individuals with severe comorbidities requiring additional treatment beyond chronic liver disease, such as hepatocellular carcinoma, were excluded because these conditions could independently affect HRQoL. In addition, individuals with a history of psychiatric disorders were excluded due to their potential confounding effects on the relationships among the study variables.

Using G*Power 3.1.5, a minimum sample size of 131 was calculated for the multiple regression analysis, with a significance level of .05, a medium effect size of .15, a power of .80, and 13 predictor variables: sex, age, spouse, educational level, occupation, monthly income, time since diagnosis of liver disease, comorbidities, absenteeism, symptom, depression, uncertainty in illness, and subjective health status. Considering a dropout rate of 20%, the target sample size was set at 164 participants. A total of 170 structured questionnaires were distributed, considering possible difficulties in survey completion among patients unfamiliar with surveys. After excluding 4 incomplete responses, 166 responses were included in final data analysis.

### 3. Instruments

#### 1) General characteristics of participants

The participants’ general characteristics included sex, age, spouse, educational level, occupation, monthly income, time since diagnosis of liver disease, comorbidities, absenteeism, and symptoms.

#### 2) HRQoL

HRQoL was measured using the World Health Organization’s WHOQoL-BREF tool [15]. The Korean version of the WHOQoL-BREF was used after obtaining permission through the WHOQoL materials permission process provided on the WHO website. The instrument contains 26 items and is structured to measure physical health, psychological health, social relationships, and environmental domains. In this study, the overall WHOQoL-BREF score was used to assess participants’ overall HRQoL. Each item uses a 5-point Likert scale, with reverse scoring applied to negatively worded questions. Higher scores indicate a higher HRQoL. The Korean version of the WHOQoL-BREF [16] reported an overall Cronbach's α of .90. In this study, Cronbach's α was .93.

#### 3) Depression

We used an instrument adapted by Cho and Kim [17] based on the Center for Epidemiologic Studies Depression Index (CES-D) developed by Radloff [18]. The CES-D is a public-domain instrument and can be used without permission. This tool, which is widely used for screening depressive symptoms, consists of 20 items, with positively worded items were reverse-scored. Each item assesses depressive symptoms experienced over the past week, scored as follows: “rarely” (0 points), “sometimes” (1 point), “often” (2 points), and “almost always” (3 points). Cronbach’s α was .89 in the study by Cho and Kim [17], and .88 in the present study.

#### 4) Uncertainty in illness

Uncertainty in illness was assessed using the tool translated into Korean by Chung et al. [19] based on Mishel’s Uncertainty in Illness Scale [20]. Permission to use the instrument was obtained from the University of North Carolina at Chapel Hill. This instrument consists of 23 items rated on a 5-point Likert scale ranging from “strongly agree” (5 points) to “strongly disagree” (1 point). Negatively worded items were reverse scored, and higher scores indicated higher levels of uncertainty. In Chung et al.’s study [19], Cronbach’s α was .85, and in the present study, it was .84.

#### 5) Subjective health status

Subjective health status was measured using the Health Self-Rating Scale adapted by Sung and Kim [21] for the Korean context and subsequently used as a three-item instrument by Shin and Kim [22], after obtaining permission for its use. The 3 items assess current self-rated health status, health status compared with one year ago, and health status compared with peers of the same age. Each item is rated on a 5-point Likert scale. Higher scores indicate better subjective health status. Cronbach’s α was .70 in the study by Shin and Kim [22] and .76 in the present study.

### 4. Data collection

Data were collected between December 2022 and February 2023 using a convenience sampling method among patients waiting for outpatient treatment for liver disease. Prior to data collection, the research assistants received training regarding the study purpose, participant recruitment procedures, ethical considerations, and standardized survey administration methods to ensure consistency in data collection. The questionnaire required approximately 10-15 minutes to complete. Participants were informed that their responses would remain anonymous and confidential and that participation in the study was voluntary. Written informed consent was obtained before participation.

### 5. Data analysis

The collected data were analyzed using SPSS version 23 (IBM Corp., Armonk, NY, USA). Participants’ general characteristics and the study variables were summarized using descriptive statistics, including frequencies, percentages, means, and standard deviations. Cronbach’s α coefficients were calculated to assess the internal consistency of the instruments. Differences in HRQoL according to participants’ general characteristics were examined using independent t-tests and one-way analysis of variance, followed by Scheffé post hoc tests when appropriate. Pearson’s correlation coefficients were calculated to examine the associations among HRQoL, depression, uncertainty in illness, and subjective health status. Multiple linear regression analysis was performed to identify factors associated with HRQoL. Prior to regression analysis, the assumptions of independence of residuals, multicollinearity, normality of residuals, and homoscedasticity were assessed.

### 6. Ethical considerations

This study was approved by the Institutional Review Board (IRB) of Presbyterian Medical Center (IRB No. 2022-10-066). The study hospital is a general hospital located in Jeonju, South Korea, providing diagnosis, treatment, and follow-up care for patients with various chronic liver diseases.

The survey was conducted after obtaining written informed consent from participants who understood the purpose of the study and voluntarily agreed to participate. The collected data were used solely for research purposes, and participants were informed that they could withdraw from the study at any time. They were encouraged to contact the researchers if they had any questions. Participants were informed that the collected data would be encrypted and securely managed, stored for 3 years after the completion of the study, and then permanently destroyed using secure methods. After completing the survey, participants received a modest gift in appreciation of their participation.

## RESULTS

### 1. Characteristics of the participants and differences in HRQoL according to characteristics of participants

Among the 166 participants, 54.8% were men and 45.2% were women. The age distribution was 7.8% aged 40 years or younger, 10.3% aged 41–49 years, 27.1% aged 50–59 years, and 54.8% were aged 60 years and older, with a mean age of 61.12 ± 13.52 years. A total of 65.1% of the participants had a spouse, whereas 34.9% did not. Educationally, 30.7% had completed middle school or below and 69.3% had completed high school or higher. In terms of occupation, 46.4% were employed while 53.6% were not. Regarding monthly income, 57.2% earned less than 2 million won, 16.9% earned 2–2.99 million won, and 25.9% earned 3 million won or more. Regarding time since diagnosis, 18.7% did not know, 16.9% were diagnosed less than 2 years ago, and 64.4% were diagnosed 2 years ago or longer. Regarding comorbidities, 18.7% of the participants had comorbidities, and 10.2% reported absenteeism due to liver disease. Symptoms were present in 31.9% of cases and absent in 68.1%.

In terms of HRQoL, those with a spouse scored 3.25 ± 0.52 points compared to 3.08 ± 0.57 points for those without, indicating a statistically significant difference (t = -2.02, *p* = .045). For those with education level of middle school graduation or below, the HRQoL score was 3.01 ± 0.53 points, whereas for those with high school graduation or above, it was 3.27 ± 0.53 points, showing that those with higher education had higher scores, with a statistically significant difference (t = -2.92, *p* = .004). In terms of monthly income, individuals earning less than 2 million won had a score of 3.06 ± 0.57 points, those earning between 2-2.99 million won had 3.39 ± 0.48 points, and those earning 3 million won or more had 3.36 ± 0.44 points. The group earning 2–2.99 million won showed the highest HRQoL scores, with a statistically significant difference (F = 7.60, *p* = .001). Post hoc analysis indicated that the scores were higher in the 2–2.99 million won and 3 million won or more groups than in those earning less than 2 million won. Participants who had absenteeism due to chronic liver disease had a score of 2.91 ± 0.51 points, whereas those without absenteeism scored 3.23 ± 0.54 points. There was a statistically significant difference in the HRQoL scores between the groups (t = 2.32, *p* = .022). Regarding symptoms, those with symptoms had a score of 2.97 ± 0.51 points, while those without symptoms had 3.29 ± 0.53 points. There was a statistically significant difference, indicating that those without symptoms had higher HRQoL scores (t = 3.68, *p* < .001). There were no significant differences in HRQoL according to participants’ sex, age, occupation, time since diagnosis, and comorbidities (Table 1).

### 2. Participants’ levels of depression, uncertainty in illness, subjective health status, and HRQoL

The level of depression was 0.88 ± 0.46 points on a scale of 0 to 3 points, uncertainty in illness was 2.70 ± 0.48 points on a scale of 1 to 5 points, subjective health status was 2.96 ± 0.66 points on a scale of 1 to 5 points, and HRQoL was 3.19 ± 0.54 points on a scale of 1 to 5 points (Table 2).

### 3. Correlations between participants’ depression, uncertainty in illness, subjective health status and HRQoL

Depression (r = -.48, *p* < .001) and uncertainty in illness (r = -.43, *p* < .001) were significantly and negatively correlated with HRQoL. Subjective health status was significantly and positively correlated with HRQoL (r = .51, *p* < .001) (Table 3).

### 4. Factors influencing participants’ HRQoL

To identify factors influencing HRQoL among the participants, spouse, education level, monthly income, absenteeism, and symptoms, which showed significant differences in HRQoL in the analysis of general characteristics, were selected as control variables and processed as dummy variables. Regression analysis was conducted after examining the independence of residuals and multicollinearity among the independent variables with Durbin-Watson statistic of 2.01 (d_*U*_ = 1.85) indicating no autocorrelation and variance inflation factor values ranging from 1.11 to 1.47, all below 10, indicating no multicollinearity. Therefore, a regression analysis was deemed appropriate.

The adequacy of the regression model was assessed by examining the normality and homoscedasticity through residual analysis. The results of the Kolmogorov-Smirnov test for standardized residuals indicated that the normality assumption was met (*p* = .114 > .05). Additionally, the Breusch-Pagan test (*p* = .128 > .05) and the Koenker test (*p* = .609 > .05) also confirmed the homoscedasticity assumption. Therefore, the regression model was deemed appropriate.

Multiple regression analysis was conducted to identify the factors influencing HRQoL among the participants. The analysis controlled for spouse, education level, monthly income, absenteeism, and symptoms. Results revealed that depression, uncertainty in illness, subjective health status, and education level were significant factors influencing HRQoL. Specifically, higher levels of depressive symptom and uncertainty in illness were associated with lower HRQoL (β = -.24, *p* = .002; β = -.15, *p* = .044, respectively), and better subjective health status was associated with higher HRQoL (β = .34, *p* < .001). Among the independent variables, subjective health status (β = .34, *p* < .001) exerted the greatest influence on HRQoL. Additionally, individuals with a higher education level (β = .17, *p* = .013) showed higher HRQoL compared to those with lower educational attainment (Table 4).

## DISCUSSION

This study aimed to identify the factors influencing the HRQoL of patients with chronic liver disease to provide foundational data for improving HRQoL and to develop effective intervention programs. According to the study findings, the HRQoL score of patients with chronic liver disease was 3.19 ± 0.54 points, which was comparable to the low HRQoL reported by Sagara et al. [23]. HRQoL in patients with chronic liver disease is a crucial variable for evaluating disease progression and treatment efficacy [23]. Given the chronic and demanding nature of the continuous treatment required for chronic liver disease, it is evident that understanding and intervening comprehensively with various factors affecting HRQoL while maintaining optimal functional status in daily life is necessary.

The HRQoL score was significantly higher among patients with spouses compared to those without spouses. Family, as the primary source of social support for individuals with chronic illness, influences various physical and psychological symptoms as well as self-care behaviors. Given that chronic liver disease requires ongoing management to prevent exacerbation, support from spouses, who are often the closest source of support for patients, can enhance adaptation to psychological difficulties and improve problem-solving abilities related to disease management. Such support may foster positive health perceptions and improve adaptation to chronic illness, ultimately contributing to better HRQoL. This interpretation is supported by previous studies reporting higher HRQoL among patients with chronic liver disease who had spouses than among those without spouses [13]. Therefore, the management of chronic liver disease should consider not only the health and well-being of individual patients but also family involvement. In particular, integrated management strategies that involve spouses as key sources of support are needed to prevent complications and delay disease progression.

In this study, educational level significantly influenced HRQoL among participants with chronic liver disease. Specifically, those with at least a high school education had significantly higher HRQoL than those with a middle school education or below. Higher educational levels can aid in coping with personal issues, while lower educational levels may lead to psychological issues and the acceptance of misinformation [24]. This finding is supported by previous research showing that higher educational levels are associated with better HRQoL among patients with chronic liver disease [23]. Therefore, it is essential to provide disease-related information tailored to the educational level of the participants and ensure psychological support through clear communication.

Monthly income was also significantly associated with HRQoL in this study. Participants earning between 2–2.99 million won per month scored significantly higher in HRQoL compared to those earning less than 2 million won per month, as did participants earning 3 million won or more. This aligns with the findings of Do et al. [13] among middle-aged male patients with chronic liver disease, in which respondents who reported sufficient monthly income had the highest HRQoL scores. Generally, higher incomes enable access to better-quality healthcare services, contribute to better health. They also reduce health risks, and alleviate concerns about future financial stability, leading to improved HRQoL [25]. Therefore, improving rational medical insurance policies to alleviate the economic burden of ongoing treatments for patients with chronic liver disease and ensuring equitable access regardless of economic status are crucial steps to support successful treatment outcomes and mitigate relative inequality based on financial circumstances.

This study investigated the impact of absenteeism and symptoms on participants’ HRQoL. Participants without absenteeism had significantly higher HRQoL scores than those with absenteeism. While there are no direct comparative studies on the differences in HRQoL scores according to absenteeism, previous research suggests that absenteeism significantly affects depression among patients with chronic liver disease [26], indicating a negative impact on HRQoL. Absenteeism affects not only individual health but also workplace productivity. Therefore, developing health promotion programs at the corporate level to delay symptom exacerbation and disease progression in patients with chronic liver disease and implementing national disease management policies are necessary.

In this study, patients with no symptoms had a higher HRQoL score than those with symptoms. This finding supports previous research among middle-aged men with chronic liver disease, which found that experiencing symptoms significantly affected HRQoL [13]. Symptoms of chronic liver disease can manifest at any stage of its natural course and are associated with various systemic symptoms that can impair patients’ quality of life [27]. Therefore, systematic and repeated education is needed to prevent symptoms and ensure appropriate treatment based on the patient's disease status.

In this study, the symptoms of depression were identified as a factor that lowers HRQoL, which is consistent with previous studies by Park and Yang [9], who found that more severe depressive symptoms were associated with lower HRQoL. The depression score in this study was slightly lower than that reported by Park and Yang [9], possibly due to advancements in medical technology and treatment options for liver disease as well as easier access to health information. However, considering research indicating higher outpatient visits and hospitalization rates among individuals with both chronic illness and depression [28], depression in patients with chronic liver disease may still contribute to increased medical costs. Depression in patients with chronic liver disease may be closely related to the unique characteristics of the disease rather than being merely an emotional problem. Patients with chronic liver disease often experience long-term treatment burden, uncertainty regarding disease progression and prognosis, and concerns about progression to cirrhosis or hepatocellular carcinoma [10,14]. In addition, social stigma associated with liver disease may lead to shame, social isolation, and psychological distress, which can interfere with patients’ adaptation to chronic illness and ultimately contribute to poorer HRQoL [29]. Therefore, continuous monitoring and psychological support for depressive symptoms are essential.

In this study, the uncertainty in illness score was 2.70 ± 0.48 points, which aligns with findings from a study on chronic hepatitis C patients [10], where uncertainty scores were higher than the midpoint. Additionally, uncertainty was identified as a significant factor influencing HRQoL among chronic liver disease patients. Uncertainty arises when individuals cannot define the meaning of illness-related events, which pose significant psychological stress [30]. Chronic liver disease, known for its difficulty in recovery and potential progression to complications such as liver cirrhosis or hepatocellular carcinoma if not managed properly, adds considerable psychological burden [14]. As uncertainty increases, coping abilities diminish, and fear often outweighs the motivation to manage the disease. Therefore, enhancing patients' ability to self-manage uncertainty by providing up-to-date information, support based on their coping styles, current disease monitoring levels, and educational backgrounds is crucial.

Subjective health status was identified as the most significant predictor of HRQoL. It is particularly important in patients with chronic liver disease because it reflects their overall appraisal of persistent symptoms, functional limitations, psychological well-being, and expectations regarding future health [11]. Patients with chronic liver disease experience not only physical problems but also uncertainty regarding disease progression and the burden of long-term treatment and disease management, which may negatively influence their perceptions of health status and ultimately lead to poorer HRQoL. This finding is supported by previous studies reporting that better subjective health status was associated with higher HRQoL among patients with chronic liver disease [12]. Therefore, improving patients’ subjective perceptions of health through symptom management, psychological support, and individualized education may contribute to enhancing HRQoL in patients with chronic liver disease.

Overall, alleviating depression and uncertainty and enhancing subjective health status through tailored interventions can significantly improve the HRQoL of patients with chronic liver disease, thus reducing the psychosocial impact on their condition and promoting better health outcomes.

In interpreting these findings, the clinical heterogeneity of chronic liver disease should be considered. Because HRQoL may differ according to disease etiology, presence of cirrhosis, disease severity, treatment status, and complications, the associations identified in this study may have been partially influenced by unmeasured clinical factors. Therefore, future studies should include detailed clinical variables to more clearly examine the relative effects of psychological, cognitive, and clinical factors on HRQoL.

This study has several limitations. Because participants were recruited using a convenience sampling method, the generalizability of the findings may be limited. In addition, excluding individuals with a history of psychiatric disorders may have resulted in selection bias and may not fully reflect the diverse characteristics of patients encountered in actual clinical settings. Furthermore, as noted above, detailed clinical characteristics of chronic liver disease were not sufficiently considered. Future research using more representative samples and comprehensive clinical information is needed to further clarify the factors influencing HRQoL among patients with chronic liver disease.

## CONCLUSION

This study is significant because it provides foundational data for the development of intervention programs aimed at improving the HRQoL of patients with chronic liver disease, considering that chronic illnesses are a major cause of death in modern society. Chronic disease management extends beyond symptom relief and disease control, ultimately aiming to improve patients’ HRQoL.

The study findings indicate that HRQoL of patients with chronic liver disease was slightly lower, suggesting that there is room for further improvement in the future. Factors influencing the HRQoL in these patients included depression, uncertainty in illness, subjective health status, and education level, with subjective health status showing the greatest influence. Based on these findings, it is essential to develop intervention programs aimed at enhancing HRQoL in patients with chronic liver disease, particularly those that address depression, reduce uncertainty in illness, improve subjective health status, and provide targeted education to enhance self-management skills. This study underscores the importance of developing intervention programs that can effectively improve the HRQoL for patients with chronic liver disease. The following recommendations are proposed based on the findings of this study.

First, we recommend conducting qualitative research to comprehensively understand the various factors influencing the HRQoL of patients with chronic liver disease as it can provide deeper insights into the subjective experiences, perceptions, and behaviors of patients, which quantitative methods alone may not fully capture.

Second, based on the factors identified in this study that influence the HRQoL of patients with chronic liver disease—specifically depression, uncertainty in illness, and subjective health status, the development of strategies and programs aimed at reducing depression and uncertainty in illness, and improving subjective health status is recommended. These interventions should be rigorously evaluated to assess their effectiveness in improving the HRQoL outcomes in this patient population.

## Figures and Tables

**Table 1.** Characteristics of the Participants and Differences in Health-related Quality of Life According to Characteristics (N = 166)

Characteristics	Categories	n (%)	M ± SD	t or F	*p* (Scheffé)
Sex	Men	91 (54.8)	3.19 ± 0.54	-0.061	.951
	Women	75 (45.2)	3.20 ± 0.55		
Age (years)	≤ 40	13 (7.8)	3.27 ± 0.77	0.26	.853
	41–49	17 (10.3)	3.27 ± 0.57		
	50–59	45 (27.1)	3.17 ± 0.53		
	≥ 60	91 (54.8)	3.18 ± 0.51		
		61.12 ± 13.52			
Spouse	Yes	108 (65.1)	3.25 ± 0.52	-2.02	.045
	No	58 (34.9)	3.08 ± 0.57		
Education level	≤ Middle school	51 (30.7)	3.01 ± 0.53	-2.92	.004
	≥ High school	115 (69.3)	3.27 ± 0.53		
Occupation	Yes	77 (46.4)	3.23 ± 0.53	-1.03	.304
	No	89 (53.6)	3.15 ± 0.55		
Monthly income (10,000 KRW)	< 200^a^	95 (57.2)	3.06 ± 0.57	7.60	.001 (a < b,c)
	200–299^b^	28 (16.9)	3.39 ± 0.48		
	≥ 300^c^	43 (25.9)	3.36 ± 0.44		
Time since diagnosis (year)	Don‘t know	31 (18.7)	3.10 ± 0.49	0.59	.557
	< 2	28 (16.9)	3.20 ± 0.57		
	≥ 2	107 (64.4)	3.22 ± 0.55		
Comorbidities	Yes	31 (18.7)	3.12 ± 0.49	-0.84	.405
	No	135 (81.3)	3.21 ± 0.56		
Absenteeism	Yes	17 (10.2)	2.91 ± 0.51	2.32	.022
	No	149 (89.8)	3.23 ± 0.54		
Symptom	Yes	53 (31.9)	2.97 ± 0.51	3.68	<.001
	No	113 (68.1)	3.29 ± 0.53		

Superscript letters (a, b, c) indicate groups compared using the Scheffé post hoc test. M = Mean; SD = Standard deviation; KRW = Korean won.

**Table 2.** Participants’ Levels of Depression, Uncertainty in Illness, Subjective Health Status, and Health-related Quality of Life (N = 166)

Variables	Possible range	M ± SD	Observed range
Depression	0–3	0.88 ± 0.46	0–3
Uncertainty in illness	1–5	2.70 ± 0.48	1–4
Subjective health status	1–5	2.96 ± 0.66	1–4
Health-related quality of life	1–5	3.19 ± 0.54	2–5

M = Mean; SD = Standard deviation.

**Table 3.** Correlations between Participants’ Depression, Uncertainty in Illness, Subjective Health Status and Health-related Quality of Life (N = 166)

Variables	Depression	Uncertainty in illness	Subjective health status	Health-related quality of life
Depression	1	-	-	-
Uncertainty in illness	.48 (<.001)	1	-	-
Subjective health status	-.39 (< .001)	-.38 (< .001)	1	-
Health-related quality of life	-.48 (< .001)	-.43 (< .001)	.51 (< .001)	1

**Table 4.** Factors Influencing Participants’ Health-related Quality of Life (N = 166)

Variables	B	β	t	*p*	VIF
(constant)	2.93		9.35	< .001	
Depression	−0.28	−.24	−3.22	.002	1.47
Uncertainty in illness	−0.17	−.15	−2.03	.044	1.44
Subjective health status	0.28	.34	4.80	< .001	1.36
Spouse^†^ (yes) (ref. no)	−0.05	−.04	−0.62	.534	1.11
Education level^†^ (≥ high school) (ref. ≤ middle school)	0.20	.17	2.51	.013	1.27
Monthly income^†^ (200–299) (ref. < 200)	0.15	.10	1.50	.135	1.25
Monthly income^†^ (≥ 300) (ref. < 200)	0.11	.09	1.33	.186	1.33
Absenteeism^†^ (yes) (ref. no)	−0.08	−.04	−0.66	.514	1.26
Symptom^†^ (yes) (ref. no)	−0.06	−.05	−0.76	.447	1.38
Adjusted R^2^ = .40, F = 13.35 (*p* < .001), Durbin-Watson’s *d* = 2.01					
Breusch-Pagan test (chi-square = 13.84, *p* = .128), Koenker test (chi-square = 7.27, *p* = .609)					
Kolmogorov-Smirnov test (z = 1.20, *p* = .114)					

VIF = Variance inflation factor; ref. = Reference.

† Dummy variables.
